# Motor outcomes in patients with advanced Parkinson’s disease treated with levodopa/carbidopa intestinal gel in Italy: an interim analysis from the GREENFIELD observational study

**DOI:** 10.1007/s10072-016-2664-0

**Published:** 2016-07-15

**Authors:** Leonardo Lopiano, Nicola Modugno, Pietro Marano, Mariachiara Sensi, Giuseppe Meco, Antonino Cannas, Graziano Gusmaroli, Filippo Tamma, Francesca Mancini, Rocco Quatrale, Anna Maria Costanzo, Giuliana Gualberti, Gabriella Melzi, Umberto di Luzio Paparatti, Angelo Antonini

**Affiliations:** 1 Department of Neuroscience, University of Torino, Torino, Italy; 2 Neurology Department, IRCCS Neuromed, Pozzilli (IS), Italy; 3 Neurorehabilitation Unit, Casa di Cura Villa dei Gerani, Catania, Italy; 4 Neurology Unit, Hospital Sant’Anna, Ferrara, Italy; 5 Department of Neurology and Psychiatry (Parkinson’s Centre) and Research Centre of Social Diseases (CIMS), Sapienza University, Rome, Italy; 6 Neurology Unit, Policlinico Universitario Monserrato, Cagliari, Italy; 7 Neurology Unit, Ospedale degli Infermi, Biella, Italy; 8 Neurology Unit, Miulli Hospital, Acquaviva delle Fonti (BA), Italy; 9 Parkinson Disease and Movement Disorders Centre, Neurology Unit, S.Pio X Clinic, Milan, Italy; 10 Neurology Unit, Hospital dell’Angelo, Mestre (VE), Italy; 11 AbbVie Srl, Campoverde (LT), Italy; 12 Parkinson and Movement Disorder Unit, IRCCS Hospital San Camillo, Venice, Italy

**Keywords:** Advanced Parkinson’s disease, Levodopa–carbidopa, Intestinal infusion, Motor symptoms, Quality of life, Routine patient care

## Abstract

Several levodopa/carbidopa intestinal gel (LCIG) studies showed a significant reduction of OFF time and a significant increase of ON time, as well as a reduction of dyskinesia, and improvement of non-motor symptoms and quality of life. However, few studies have been conducted in a large population for more than 3 years. Interim outcomes from GREENFIELD observational study on a large Italian cohort of advanced PD patients who started LCIG in routine care between 2007 and 2014, still on treatment at the enrollment, are presented. Comparison between baseline (before LCIG start) and visit 1 (at enrollment) is reported. Primary endpoint was Unified Parkinson’s Disease Rating Scale (UPDRS) IV Item 39; secondary endpoints were UPDRS I and II, as outcome of quality of life. Overall, 145 of 148 enrolled patients from 14 Movement Disorder Centers in Italy were evaluable with a mean LCIG treatment period of 1.38 ± 1.66 years at enrollment. Compared with baseline, the mean score regarding daily time spent in OFF (UPDRS IV Item 39) at visit 1 significantly decreased from 2.1 ± 0.8 to 0.9 ± 0.7 (57 % reduction vs baseline, *P* < 0.0001); UPDRS IV improved by 39 % (*P* < 0.0001); scores for dyskinesia duration and disability were reduced by 28 % (1.8 ± 1.0–1.3 ± 0.9; *P* < 0.0001) and 33 % (1.5 ± 1.1 to 1.0 ± 1.0; *P* < 0.0001), respectively; and the scores for painful dyskinesia and early morning dystonia were reduced by 56 % (0.9 ± 1.0–0.4 ± 0.7; *P* < 0.0001) and 25 % (0.4 ± 0.5–0.3 ± 0.5; *P* < 0.001), respectively. The preliminary results of this interim analysis support the efficacy of LCIG on motor complications and activities of daily living.

## Introduction

Parkinson’s disease (PD) is a chronic, progressive neurodegenerative disorder characterized by motor impairments (tremor, rigidity, bradykinesia, and postural instability) [1]. Further features include non-motor symptoms, such as cognitive dysfunction, depression, and sleep disorders [1, 2], resulting in reduced quality of life [3] and negative effects on social interactions [3, 4]. Moreover, patients with PD have a progressive loss of autonomy, with a consequent impact on caregiver quality of life.

As the disease progresses, the response duration to levodopa shortens and the therapeutic window narrows, resulting in unpredictable fluctuations, with random and sudden ‘‘OFF’’ periods, as well as disabling dyskinesia, which exert a negative impact on the overall daily activities and quality of life [5]. Motor and non-motor symptoms reflect fluctuations in levodopa plasma concentrations due to the short half-life of levodopa and erratic absorption in relation with delayed gastric emptying [6].

Continuous dopaminergic drug delivery, obtained with the administration of intraduodenal levodopa/carbidopa intestinal gel (LCIG), has been shown to provide a more stable plasma concentration of levodopa in patients with non-optimal control of motor fluctuations [7]. A number of studies have shown that LCIG leads to a significant reduction of OFF time and a significant increase of ON time, as well as a reduction of dyskinesias [8–11]. In addition, improvements in non-motor symptoms—and quality of life—were observed [12, 13]. However, few studies have been conducted in a large population of patients with PD to assess the long-term outcome (over a period of >2 years) of treatment with LCIG [14, 15]. Therefore, the aim of this observational study was to evaluate the clinical outcomes of a large Italian cohort of patients with advanced PD receiving LCIG in routine clinical care to evaluate the effects of therapy on both motor and non-motor symptoms and the related impact on patient quality of life and caregiver burden from the initiation of LCIG therapy over a maximum exposure period of up to 9 years. Here, we present the interim results on motor symptoms and Unified Parkinson’s Disease Rating Scale (UPDRS) scores in this large cohort of patients with advanced PD.

## Patients and methods

### Study design

This observational study was conducted in 14 movement disorder centers throughout the Italian territory.

Treatment with LCIG was initiated in a routine patient care setting, according to the Summary of Product Characteristics, including the nasointestinal phase.

### Patient selection

Consecutive patients with advanced PD and motor complications, who started LCIG infusion according to clinical practice between 2007 and 2014, were considered for enrollment into the study.

Inclusion criteria were being treated with LCIG, the presence of adequate information about the previous medical history and treatment, and the presence of at least one fulfilled scale or questionnaire among a selected list. Patients could be enrolled at any time after LCIG treatment initiation. Exclusion criteria were the presence of conditions that could have interfered with the long-term continuation of LCIG therapy at the physician’s discretion.

Patients fulfilling inclusion criteria were enrolled in the study at visit 1; during this visit, patient history and retrospective clinical parameters referred to the previous conventional PD treatment, nasointestinal phase, and initiation of LCIG treatment via PEG-J were collected as baseline (BL) data. During the same visit, the current clinical parameters were also collected as Visit 1 data. For the analysis, BL was defined as the last available data collected prior to NJ tube positioning.

The study design included two patient populations: the retrospective population and the prospective population. The retrospective population includes all patients who had been receiving treatment with LCIG for >1 year and up to 7 years before the enrollment visit (visit 1), with available BL retrospective assessment data for >1 year. The prospective population includes all patients receiving treatment with LCIG for <1 year before the enrollment visit. Patients continuing with LCIG treatment for further 2 years after enrolment and with follow-up visits on yearly basis will be included in the final analysis. Here, we present the interim results on data collected at Visit 1 on the overall population.

The protocol of the study was approved by the Ethics Committee of each local health authority. Each patient provided informed consent. The study was conducted according to the International Conference on Harmonization Good Clinical Practices.

### Patient evaluation

For the interim analysis at Visit 1 (enrolment), the following assessments were considered:

- BASELINE data, including demographic characteristics, medical history, previous PD treatments, nasointestinal phase, LCIG treatment doses, including the total daily dose of infusion at discharge from the hospital, the Hoehn and Yahr scale, and the UPDRS I, II, and IV if available.
- VISIT 1 data, including the LCIG treatment doses, Hoehn and Yahr scale, and the UPDRS I, II, and IV.

The primary endpoint of this study was the Item 39 of the UPDRS IV (percentage of waking day spent in OFF) at the last available follow-up compared with BL. For the interim analysis, the comparison between visit 1 assessment and BL data was analysed, as described in the protocol.

Secondary effectiveness measures included UPDRS I total score (in ON and OFF conditions), and activities of daily living (ADL), as assessed by means of the UPDRS II (in ON and OFF conditions). Motor complications were assessed by means of the UPDRS IV total score and subitems for dyskinesia duration (Item 32), dyskinesia severity (Item 33), painful dyskinesia (Item 34), and early morning dystonia (Item 35). Safety data will be analysed at study closure, since they were collected from enrolment visit onward. For this reason, in this interim investigation, no adverse events have been included.

### Statistical analysis

Quantitative variables were summarized using the number of non-missing observations, mean, standard deviation (SD), median, first and third quartile, minimum, and maximum. Categorical variables were summarized using frequency count and percentage distribution. Statistical significance was considered to be met when the rounded *P* was less than ≤0.05. Comparison between BL and the last follow-up values of all endpoints were performed using a Wilcoxon signed-rank test.

## Results

The first patient was enrolled in November 2012; through July 2014, a total of 148 patients were included among the participating centers. Three subjects were excluded from the evaluable population, as they violated the inclusion/exclusion criteria.

Demographic characteristics, medical history, occupational status, and PD features are summarized in Table 1. Economical and aids supports for patients supplied by the Italian Healthcare System are reported in Table 1. The mean age (mean ± SD) of patients was 70.4 ± 7.7 years (with 79.3 % of the population aged over 65 years), the mean duration of PD was 14.6 ± 6.6 years, and the mean time since the onset of motor fluctuations was 5.9 ± 4.0 years.

**Table 1:** Demographic and clinical characteristics of the study population

Parameter	Value	Range
Demographics	*n* = 145	
Mean ± SD age, years	70.4 ± 7.7	49–90
Age		
<65 years, *n* (%)	30 (20.7 %)	
≥65 years, *n* (%)	115 (79.3 %)	
Age >70 years, n (%)	78 (53.8 %)	
Females, *n* (%)	72 (49.7 %)	
Males, *n* (%)	73 (50.3 %)	
White race, *n* (%)	144 (99 %)	
Mean ± SD height, cm	164.4 ± 8.5	145–185
Occupational status		
Worker, *n* (%)	5 (3.4 %)	
Retired, *n* (%)	116 (80 %)	
Housekeeper, *n* (%)	11 (7.6 %)	
Unemployed, *n* (%)	13 (9 %)	
PD medical history		
Mean ± SD age at PD diagnosis, years	55.7 ± 0.77	
Mean ± SD PD duration at visit 1, years	14.61 ± 6.58	1.3–46.7
Mean ± SD time since onset of motor fluctuations at visit 1, years (*n* = 143)	5.9 ± 4.0	1–21
LCIG duration at enrollment, *n* (%)		
≤1 year	105 (72.4 %)	
1–3 years	19 (13.1 %)	
≥3 years	21 (14.5 %)	
Previous antiparkinsonian treatments (before LCIG infusion)	*N* (%)	Daily dose, mean ± SD
Previous deep brain stimulation	3 (2.1 %)	NA
Apomorphine SC (pump) (mg)	14 (9.7 %)	86.29 (46.38)
Apomorphine stylo (mg)	7 (4.8 %)	6.5 (10.6)
Support by the NHS	*N* (%)	
NHS payment because of PD	101 (69.7 %)	
Care family allowance	81 (55.9 %)	
Disability pension	79 (54.5 %)	
Use of aids supplied by NHS	41 (28.3 %)	

*LCIG* levodopa/carbidopa intestinal gel, *NHS* National Health Service, *PD* Parkinson’s disease, *SC* subcutaneous, *SD* standard deviation

Previous antiparkinsonian medications before the initiation of LCIG infusion and the corresponding mean daily dosages are reported in Table 2. At the start of LCIG infusion, oral levodopa was the most commonly used antiparkinsonian medication (96.6 % of patients, at a mean daily dose of 812.17 ± 409.9 mg), followed by dopamine agonists (64.1 %). The use of antiparkinsonian medications after LCIG initiation was largely reduced, as reported in Table 2. The primary reasons for the initiation of LCIG treatment were disabling OFF periods in 111 patients (76.6 %) and uncontrolled dyskinesia in 29 patients (20 %).

**Table 2:** Use of antiparkinsonian medications before and during LCIG at visit 1 among the 145 evaluable patients

Antiparkinsonian medications	Before LCIG start		At visit 1	
	*N* (%)	Daily dose, mean ± SD	*N* (%)	Daily dose, mean ± SD
Oral levodopa (mg)	140 (96.6 %)	812.17 ± 409.93	7 (5 %)—during the day 37 (26 %)—at night	307.0 ± 281.0 during the day 155.4 ± 75.3 at night
Dopamine agonists (mg)	93 (64.1 %)	6.38 ± 5.6	44 (30 %)	5.6 ± 3.8
COMT inhibitors (mg)	64 (44.1 %)	577.8 ± 336.8	17 (12 %)	255.9 ± 102.9
MAO inhibitors (mg)	21 (14.5 %)	2.33 ± 3.31	5 (3 %)	3.6 ± 4.0
Amantadine (mg)	25 (17.2 %)	190.6 ± 112.6	8 (6 %)	237.5 ± 91.6

*COMT* catechol-O-methyl transferase, *MAO* monoamine oxidase, *NA* not available, *SD* standard deviation

At visit 1, the mean LCIG duration was 1.38 ± 1.66 years; the mean duration of LCIG infusion per day was 13.55 ± 3.05 h during daytime and was terminated at bedtime in all patients; and the infusion duration was similar at the discharge from the hospital after nasointestinal titration (13.23 ± 3.4 h). The mean duration of LCIG treatment at the time of the enrollment in the study was 1.38 ± 1.66 years (median value 0.79), with 28 % of the patients receiving LCIG infusion for at least 2 years (Table 1). The mean total continuous infusion dose at LCIG start was 3.34 ± 1.22 ml/h, remaining stable at visit 1 (3.21 ± 1.09 ml/h). The average morning dose was 8.78 ± 3.4 ml at LCIG initiation and 9.08 ml at visit 1 (including 3 ml for filling the device). At LCIG initiation, a mean of 1.5 ± 1.3 extra bolus doses was administered to 95 % of the patients, and this number remained constant at Visit 1 (1.6 ± 1.2, in 100 % of the patients).

**Fig. 1:**
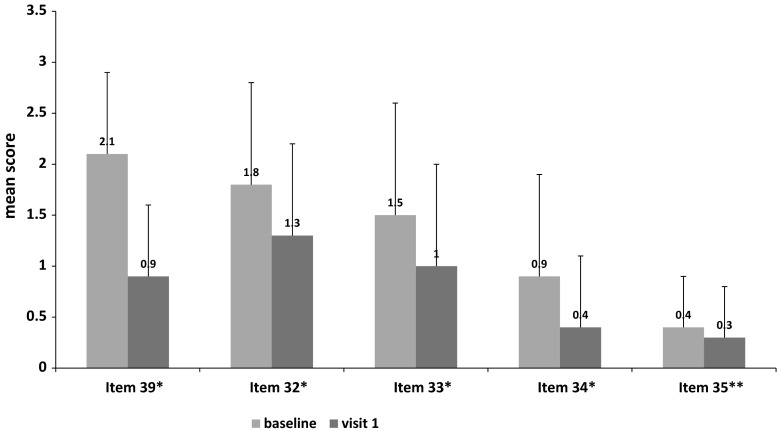
UPDRS-Part IV mean subscores ± SD at baseline (under conventional standard treatment) and at visit 1 (under LCIG treatment); *asterisks* represent statistical significance (*P < 0.0001), **P = 0.0002) compared to baseline from paired *t* test. *UPDRS* Unified Parkinson’s Disease Rating Scale

**Fig. 2:**
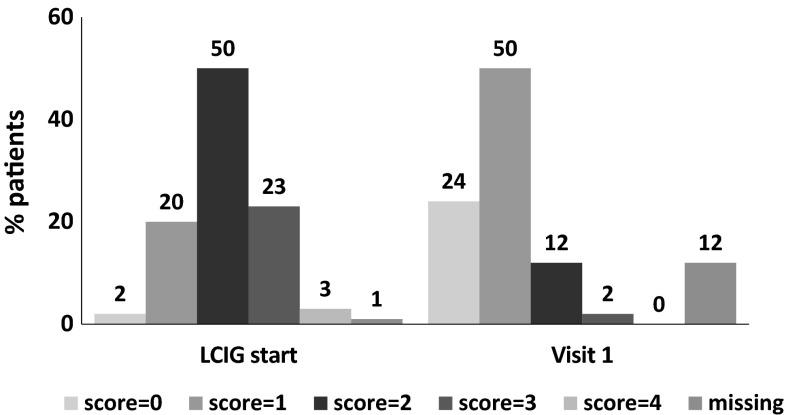
Proportion of waking day spent in OFF state according to UPDRS-Part IV Item 39 (0 = none; 1 = 1–25 % of day; 2 = 26–50 % of day; 3 = 51–75 % of day; 4 = 76–100 % of day)

Compared with BL, the mean score for daily OFF time (UPDRS IV Item 39; assessed in 88 % of the patients at visit 1) significantly decreased from 2.1 ± 0.8 to 0.9 ± 0.7, with a reduction of 1.2 points (57 % reduction compared with BL, *P* < 0.0001; Fig. 1). Moreover, 74 % of the patients at visit 1 showed an UPDRS IV Item 39 score ranged 0 or 1 (Fig. 2).

Baseline assessments of motor complications in patients receiving conventional PD treatment before the initiation of LCIG infusion were collected at visit 1 and are presented in Table 3.

**Table 3:** Complications of therapy (UPDRS IV) at baseline (before LCIG treatment) and after a mean LCIG treatment period of 1.38 ± 1.66 years (visit 1)

	BL mean score (±SD)	*N*	Range	Visit 1 mean score (±SD)	*N*	Range	Reduction vs BL (%)	*P* value
UPDRS IV total score (items 32–42)	8.5 (3.4)	138	0–18	5.2 (4.2)	126	0–34	39	<0.0001
dyskinesia duration (item 32)	1.8 (1.0)	142	0–4	1.3 (0.9)	128	0–4	28	<0.0001
dyskinesia disability (item 33)	1.5 (1.1)	141	0–4	1.0 (1.0)	127	0–4	33	<0.0001
dyskinesia pain (item 34)	0.9 (1.0)	141	0–4	0.4 (0.7)	127	0–4	56	<0.0001
early morning dystonia (item 35)	0.4 (0.5)	141	0–1	0.3 (0.5)	128	0–1	25	0.0002
OFF time duration (item 39)	2.1 (0.8)	143	0–4	0.9 (0.7)	128	0–3	57	<0.0001
UPDRS I total score								
OFF	6.9 (4.7)	87	0–16	5.6 (4.0)	73	0–15	19	<0.0001
ON	4.5 (3.1)	103	0–12	3.6 (2.8)	121	0–12	20	0.0191
UPDRS II (ADL) total score								
OFF	29.5 (9.9)	105	0–52	25.8 (10.2)	82	3–50	13	<0.0001
ON	18.6 (9.5)	118	0–39	17.0 (8.9)	126	0–44	9	0.0033
UPDRS V (Hoehn and Yahr) total score								
OFF	4.0 (0.8)	128	2–5	3.6 (0.9)	117	0–5	10	<0.0001
ON	3.1 (0.8)	143	1–5	2.8 (0.8)	145	1–5	10	<0.0001

*ADL* activities of daily living, *BL* baseline, *LCIG* levodopa/carbidopa intestinal gel, *SD* standard deviation, *UPDRS* United Parkinson’s Disease Rating Scale

Compared with BL, complications of therapy, as assessed by the UPDRS IV score and improved by 39 % (*P* < 0.0001); the UPDRS IV Item 32 score for dyskinesia duration was reduced by 28 % (1.8 ± 1.0–1.3 ± 0.9; *P* < 0.0001); the UPDRS IV Item 33 score for dyskinesia disability was reduced by 33 % (1.5 ± 1.1–1.0 ± 1.0; *P* < 0.0001); the UPDRS IV Item 34 score for painful dyskinesia was reduced by 56 % (0.9 ± 1.0–0.4 ± 0.7; *P* < 0.0001); and the UPDRS IV Item 35 score for early morning dystonia was reduced by 25 % (0.4 ± 0.5–0.3 ± 0.5; *P* < 0.001; Table 3).

Regarding the efficacy measures commonly associated with cognitive function and quality of life in ADL, significant improvement was observed in UPDRS I and UPDRS II scores. Compared with BL, the mean change for UPDRS I was 1.3 points in OFF and 0.9 in ON (−19 and −20 %, respectively), while the mean change for UPDRS II was 3.7 points in OFF and 1.6 in ON (−13 and −9 %, respectively; Table 3).

## Discussion

Here, we report results from the largest Italian cohort of patients with advanced PD treated with LCIG in routine clinical practice, with patients from 14 Movement Disorder Centers. The population enrolled in this study was represented by advanced PD patients with motor fluctuations and dyskinesias not optimally controlled by conventional oral and transdermal treatments. The interim analysis showed a significant reduction in total daily OFF time after a mean of 1.4 years of LCIG use; the magnitude of improvement was consistent with the results reported in the previous studies [15–17]. Moreover, the high percentage of patients reporting a UPDRS item-39 score of 0 or 1 of Item 39 of the UPDRS IV (74 % of the cases) during LCIG infusion was clinically significant compared with the percentage reported under conventional treatments (22 % of the cases). The clinical relevance of this finding is further supported by the significant improvement of all the UPDRS IV items related to dyskinesias and the total score of UPDRS IV.

The results of the previous clinical studies on LCIG infusion have already indicated that this is an effective therapeutic strategy for improvements in motor symptoms (reduction in OFF time, increase in ON time without disabling dyskinesia, and reduction of troublesome dyskinesia) [8, 10], non-motor symptoms (somnolence, fatigue, cardiovascular, and urinary function) [12, 18–20], and quality of life. Recently, a 12-month interim analysis of an observational, routine care trial studying the long-term efficacy and safety of LCIG has shown significant reduction in mean daily OFF time (−4.7 h vs BL) and ON time with dyskinesia (−1.7 h vs BL), as well as a significant improvement in non-motor symptoms and quality of life [21].

Similarly, the improvements obtained in the UPDRS I and II for ADL were comparable to those reported in an international, 54-week, and open-label study in 354 patients with APD with ≥3 h per day of OFF time despite optimized therapy. In this study, the mean daily OFF time decreased by 4.4 h (65.6 %; *P* < 0.001) and ON time without troublesome dyskinesia increased by 4.8 h (62.9 %; *P* < 0.001), while ON time with troublesome dyskinesia decreased by 0.4 h (22.5 %; *P* = 0.023) [19].

Moreover, a recently published 12-week, randomized, controlled, double-blind, double-dummy trial in 71 patients with advanced PD whose motor complications were not adequately controlled (≥3 h/day OFF time) by the standard oral and transdermal therapy showed that LCIG produced 4.04 h of improvement in mean daily OFF time compared with BL and 1.91 h more than the improvement obtained with immediate-release oral levodopa-carbidopa (LC-IR) treatment. In addition, an increase of 4.11 h in mean daily ON time without troublesome dyskinesia, corresponding to 1.86 h more than the improvement seen with LC-IR treatment (95 % CI 0.56–3.17; *P* = 0.0059), was reported [22]. This beneficial effect has been confirmed in the 52 weeks open-label extension of this study on 62 patients suggesting that sustained improvement can be obtained with LCIG and that this benefit persists through 1 year of treatment [20]. This aspect is particularly relevant for LCIG long-term use, considering that due to the progressive nature of the disease, a conventional treatment could require frequent adjustments, while LCIG would represent a simplification of PD management in advanced stage.

Moreover, compared with previously published studies, recent reports in the literature cite an increase in the percentage of patients aged <65 years initiating LCIG therapy [19, 22]. Indeed, age at treatment initiation is another important aspect in LCIG selection criteria consideration: in a prospective, open-label study in 28 patients with advanced PD treated with LCIG for a mean treatment period of 24 months, younger age at operation, and the absence or mild presence of psychiatric/behavioral symptoms were positive predictive factors in selecting the best candidates for LCIG therapy [16].

This is the first Italian study with data from a large population followed for a long period of time. Since this investigation is being conducted as an observational study, with the collection of data recorded during routine medical care, we consider these outcomes to be close to “real world” clinical practice. In general, these interim outcomes are consistent with those generated in controlled short-term clinical studies. The results reported here were derived from a mean treatment period of 14 months; clinical outcomes will be followed through 24 months of follow-up in this cohort of 145 patients with advanced PD to assess the benefits of LCIG infusion therapy for up to 9 years of treatment. The possible influence of treatment duration on the motor outcome and quality of life, as well as the sub-analysis on retrospective and prospective arm, will be assessed at the end of the study.

A limitation of this interim presentation is that only data on motor complications and UPDRS II are currently available. Results on non-motor symptoms, axial symptoms, quality of life, and caregiver burden will be available in the final sample assessment. Another limitation of this study is the fact that the results are not corrected for the Levodopa Equivalent Daily Dose of concomitant oral/transdermal antiparkinsonian medications. The absence of interim data on adverse events associated with LCIG limits the ability to interpret the full benefit-risk profile in these patients and will be fully described in the final analyses.

In conclusion, these interim results confirm that treatment with LCIG produces clinically significant improvements on motor function in patients with motor symptoms not optimally controlled by oral/transdermal therapies.

## The GREENFIELD study investigators

Paolo Barone (Salerno), Annarita Bentivoglio (Roma), Antonino Cannas (Cagliari), Roberto Eleopra (Udine), Graziano Gusmaroli (Biella), Leonardo Lopiano (Torino), Francesca Mancini (Milano), Pietro Marano (Catania), Giuseppe Meco (Roma), Nicola Modugno (Pozzilli-IS), Claudio Pacchetti (Pavia), Rocco Quatrale (Mestre VE), Mariachiara Sensi (Ferrara), and Filippo Tamma (Acquaviva delle Fonti BA).
